# Trends in overweight, obesity, and waist-to-height ratio among Australian children from linguistically diverse backgrounds, 1997 to 2015

**DOI:** 10.1038/s41366-018-0139-5

**Published:** 2018-07-06

**Authors:** Louise L. Hardy, Kai Jin, Seema Mihrshahi, Ding Ding

**Affiliations:** 1 Prevention Research Collaboration, Sydney School of Public Health, University of Sydney, Sydney, NSW Australia; 2 Charles Perkins Centre, University of Sydney, Sydney, NSW Australia; 3 Sydney Nursing School, University of Sydney, Sydney, NSW Australia

**Keywords:** Risk factors, Epidemiology

## Abstract

**Objective:**

To report the cross-sectional prevalence and 18-year trends in overweight, obesity and abdominal obesity among Australian children from culturally and linguistically diverse (CALD) backgrounds.

**Subjects/Methods:**

Four cross-sectional population health surveys conducted among children (age 4-16 years; n=26, 449) in 1997-2004-2010-2015 in New South Wales, (NSW) Australia. Adiposity outcomes were measured by trained field staff using standard procedures. Binomial regression models with a robust error variance were used to estimate prevalence ratio (PR) and 95% confidence intervals (CI) for overweight and obesity, obesity, and waist-to-height ratio (WHtR) ≥ 0.5 for children from Asian, European, and Middle Eastern language backgrounds compared with children from English-speaking backgrounds, adjusted for sociodemographic characteristics.

**Results:**

Over time, children from Middle Eastern language backgrounds were consistently more likely to be overweight-obese (PR: 1.29–1.42), obese (PR: 1.49–1.65), and have WHtR ≥ 0.5 (PR: 1.42–1.90), compared with children from English-speaking backgrounds. Children from European language backgrounds generally had higher prevalence and children from Asian language backgrounds had lower prevalence, compared with children from English-speaking backgrounds. Between 1997 and 2015, there were significant trends in the prevalence of overweight and obesity combined among children from English-speaking (PR: 1.06, 95%CI: 1.02, 1.09), Middle Eastern (PR: 1.14, 95%CI: 1.05, 1.24), and Asian language backgrounds (PR: 1.14, 95%CI: 1.05, 1.24). The prevalence of WHtr ≥ 0.5 increased among children from English-speaking (PR: 1.21, 95%CI: 1.13, 1.31) and Middle Eastern (PR: 1.35, 95%CI: 1.16, 1.56) language backgrounds.

**Conclusions:**

Overall, the prevalence of overweight and obesity and abdominal obesity is high among NSW children from CALD backgrounds and has increased over time. This suggests that there is a greater scope in understanding, developing, and implementing interventions across the early life-course of children from CALD backgrounds.

## Introduction

The high prevalence of child obesity remains a global public health concern, but appears to be plateauing in many high-income countries, including Australia [1]. In Australia, sustained multi-level public health interventions have contributed to the recent stabilising of child obesity, but not among children from socially disadvantaged areas [2] and children from culturally and linguistically diverse communities (CALD) [3–5], where the prevalence of obesity is disproportionally higher. Inequalities in the population distribution of child obesity indicate obesity prevention interventions have not been effective across all population groups, which may contribute to widening health inequalities in the community.

A range of factors may influence the adiposity of children from CALD backgrounds including genetics, cultural, and social norms towards weight status, dietary practices and physical activity participation, and length of residency [6]. The interactions between these, and other factors, are complex and potentially differ across CALD communities, which may be a plausible explanation why obesity prevention interventions have not been successful in attenuating the prevalence of adiposity in children from CALD backgrounds.

The life course approach [7] to child obesity identifies preconception and pregnancy; infancy and early childhood; and older childhood and adolescence as three critical time periods for developing obesity [8]. One explanation for the higher adiposity rates among children from CALD backgrounds may be greater exposure to obesogenic factors during these critical time periods [9]. Australia is a nation with high rates of immigration and a recent government inquiry reported that people from CALD backgrounds face a disproportionate disadvantage on many social indicators. Newly arrived immigrants, particularly those with insufficient English, were more vulnerable to poverty and disadvantage, and this is reflected in their high rates of unemployment and lower health literacy and awareness of available services [10].

Over time, the cultural and language diversity of Australia has increased [11]. Twenty years ago, 15% of Australians spoke a language other than English at home, and in 2016, almost half the population were born overseas or have at least one parent born overseas and 21% speak a language other than English at home [12]. In Australia, language spoken at home is a recognised indicator of CALD background to define ‘active ethnicity’ and those who speak a non-English language at home tend to be recent immigrants [13].

In a multi-cultural society like Australia, it is important to consider CALD backgrounds in the population-level monitoring of child obesity rates to inform priority setting and decision making. However, to date, no studies have examined the temporal trends in adiposity outcomes among Australian children from CALD backgrounds. The purpose of this study was to present the prevalence differences and trends of adiposity outcomes among children from CALD backings living in New South Wales (NSW), Australia between 1997 and 2015.

## Methods

The data come from a series of NSW school-based cross-sectional population child health surveys conducted in 1997, 2004, 2010, and 2015 [14–17]. The sampling frame, sampling, and measurement protocols have remained consistent over time. Sample sizes for each survey were based on detecting a 10% group difference with 80% power and an alpha level of 0.05. All surveys were school-based and used comparable sampling frames that were based on a two-stage probability sample (school and student). The sampling frame comprised all NSW primary and high schools with the exception of special schools (e.g. blind, sport) and schools with small enrolments (i.e. <180 students). Within each sector, the schools were ordered by location (based on geo-location codes to identify rural and urban schools), gender, socio-economic status (SES), and school size. The sample of schools was therefore representative of the sector (government, independent, Catholic schools), location (rural and urban), gender composition, and SES. Schools were randomly sampled from each education sector proportional to enrolment in that sector, and students from one to two randomly selected classes in each target year were invited to participate. The surveys were conducted between February and March. Each survey was approved by the University of Sydney Human Research Ethics Committee, the NSW Department of Education, and the NSW Catholic Education Commission, and written consent by students and their carer were required for participation.

Height (m), weight (kg), and waist circumference (cm) were measured in socks, over one layer of light clothing by trained field staff using standard procedures [18]. Waist circumference was measured at the level of the narrowest point between the lower intercostal border and iliac crest with a steel anthropometric tape measure. Body mass index (BMI; kg/m^2^) was calculated from height and weight, and children were categorised as overweight and obese using the International Obesity Taskforce age–sex adjusted cut-points [19]. Waist-to-height ratio (WHtR), a proxy for abdominal obesity and predictor for cardiometabolic risk [20, 21], was calculated as waist circumference divided by height and dichotomised as a ratio < or ≥0.5 [22].

Children’s sociodemographic information collected at each survey included sex, date of birth, language spoken most at home, and postcode of residence. CALD backgrounds were classified according to participants response to the Australian Census question ‘*what language is spoken most at home*’. Response options were English or to report another language. Language spoken most at home was classified into four main language groups in NSW using the Australian Bureau of Statistics’ Australian System for Classification of Languages [23]. English-speaking; Asian based on South, Southeast, and Eastern Asian languages (e.g. Chinese, Japanese); European based on Northern, Southern, and Eastern European languages (e.g. German, Italian, Russian), and Middle Eastern based on Southwest and Central Asian languages (e.g. Arabic, Persian). Overall, approximately 1.3% (*n* = 333) children had missing language backgrounds or a language background too rare for analysis (e.g. Pacific Islands, Africa). These children were excluded from the analyses.

Children’s home postcode was used to derive a proxy measure of SES using the Australian Bureau of Statistics’ Socioeconomic Index for Areas (SEIFA) Index of Relative Socioeconomic Disadvantage score. NSW SEIFA scores were ranked into tertiles and each child’s home postcode used to determine children’s SES as low, middle, or high [24]. Postcode of residence was also used to categorise children’s geographic residence as urban (major cities) or rural (all other areas) using the Australian Bureau of Statistics’ Accessibility/Remoteness Index of Australia [25].

### Statistical analyses

Data were analysed using Stata version 14.1 in October 2017. The unadjusted prevalence of overweight and obesity combined, obesity, and WtHR ≥ 0.5 by survey year were calculated using the *svy* command to take into account the survey design and the clustering of data by school sector and school. To explore whether there were differences in the distribution of adiposity outcomes by language background at each survey point, we used log-binomial regression models with a robust error variance to estimate the prevalence ratio (PR) and 95% confidence intervals (CI) for overweight or obese combined, obese, and WHtR ≥ 0.5 by children’s language background, using English-speaking as the reference group. Survey year, sex, SES tertile, residence (urban/rural) were modelled as nominal factors and age as a continuous variable. Similarly, generalised linear models (GLM) were used to test temporal trends in adiposity outcomes by language background controlling for sociodemographic characteristics.

## Results

The response rates and sociodemographic characteristics of the children by survey year are provided in Table 1 and 2. In total, 26,449 children participated across survey years. BMI data were available for 97.6% of children and WHtR were available for 94.2% of children. The mean age across survey years ranged from 11.2 years (1997) to 10.3 years (2015), and girls comprised approximately half the survey samples. Information on the distribution of CALD communities for NSW and Australia were not available in 1997, however for the rest of survey years, the proportions of children from CALD communities were similar to the NSW and Australian CALD population of children aged 5–16 years based on language spoken most at home estimated from the most proximal Census to the survey year [26]. The proportion of children from low SES neighbourhoods declined over survey years, and a higher proportion of children from high SES neighbourhoods participated in 2015 than in 1997.

**Table 1:** Summary characteristics of the sample by survey year

	Survey year			
	1997	2004	2010	2015
Child response rates (%)	87	66	57	63
Sample size	5518	5407	8058	7555
Available BMI data (*n*)	5402	5395	7734	7374
Available WHtR data (*n*)	4391	5399	7850	7354
Girls (%)	46.5	48.9	48.0	51.5
*Age years, (mean, SD)*	11.2 (2.8)	10.5 (3.4)	10.6 (3.4)	10.3 (3.3)
*Socioeconomic status (%)*				
Low	32.8	28.1	25.9	24.5
Middle	33.5	34.8	41.3	34.4
High	33.8	37.1	32.7	41.1

*BMI* body mass index, *WHtR* waist-to-height ratio, *n/a* data not available

1997 survey: 1996 Census; 2004 survey: 2006 Census; 2010 survey:  2011 Census; and 2015 survey: 2016 Census.

**Table 2:** Language background of children age 4–16 years by survey and in NSW and Australia

	1997			2004			2010			2015		
*Linguistic background (%)*	*Population* ^a^			*Population* ^a^			*Population* ^a^			*Population* ^a^		
	Sample	*NSW*	*Aust*	Sample	*NSW*	*Aust*	Sample	*NSW*	*Aust*	Sample	*NSW*	*Aust*
English-speaking	81.9	*n/a*	*n/a*	85.9	*78.1*	*82.7*	83.2	*77.3*	*81.4*	86.0	*78.1*	*78.5*
European	4.2	*n/a*	*n/a*	2.8	*3.6*	*3.4*	1.4	*3.6*	*3.4*	1.4	*3.6*	*3.3*
Middle Eastern	4.5	*n/a*	*n/a*	3.5	*4.4*	*2.5*	4.6	*4.6*	*2.7*	4.0	*4.9*	*2.9*
Asian	7.1	*n/a*	*n/a*	7.2	*8.2*	*5.8*	9.1	*9.3*	*7.2*	7.0	*7.5*	*9.2*
Other (includes missing)	2.3	*n/a*	*n/a*	0.7	*5.7*	*5.6*	1.7	*5.1*	*5.3*	1.6	*5.9*	*6.1*

^a^Sample = survey, NSW and Australian population for children aged 4–16 years based on the most proximal Australian Census data for language spoken most at home; 1997 survey =1996 Census, 2004 survey = 2006 Census, 2010 survey = 2011 Census; and 2015 = 2016 Census.

### Adiposity outcomes by language background in each survey year

**Fig. 1:**
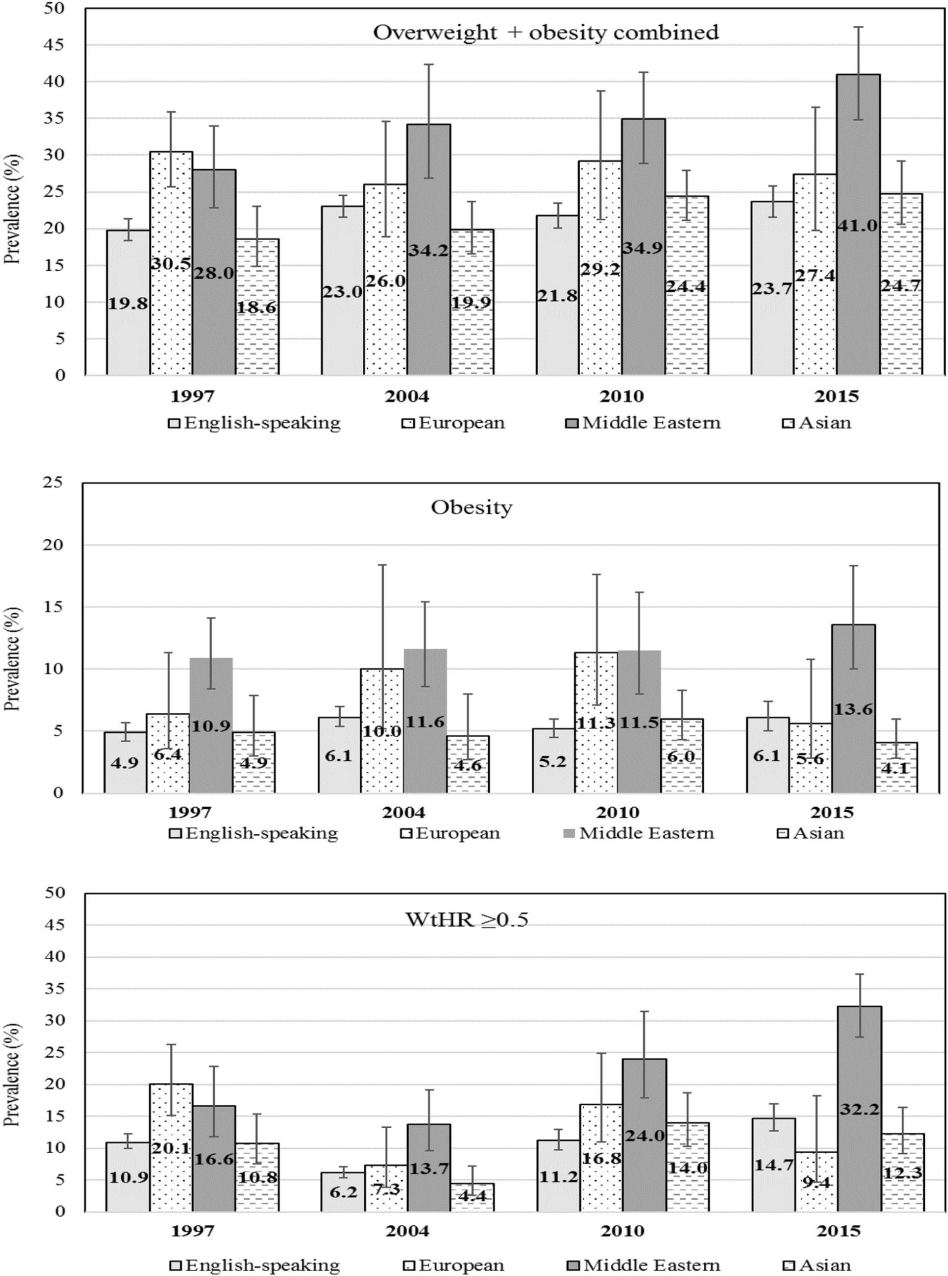
Unadjusted prevalence (%) of adiposity outcomes by language background and survey year, Australia; 1997–2015, error bars show 95%CI around the prevalence

The unadjusted point estimates of prevalence for each adiposity outcome by language background and survey year are shown in Fig. 1. The differences in adiposity outcomes by language background for each survey year are presented as PR (95%CI) with children from English-speaking backgrounds as the reference group (Table 3). Within each survey year, the prevalence of adiposity outcomes was consistently and significantly higher among children from Middle Eastern backgrounds. A less consistent pattern of higher prevalence of adiposity outcomes was observed among children from European backgrounds. For children from Asian backgrounds, the risk of adiposity outcomes was generally lower than children from English-speaking backgrounds, albeit this was only statistically significant for obesity in 2015.

**Table 3:** Within year adjusted prevalence ratio (PR, 95%CI) of adiposity outcomes by linguistic background (reference group is English-speaking), Australia; 1997–2015

Adiposity outcomes	1997	2004	2010	2015
	PR (95%CI)	PR (95%CI)	PR (95%CI)	PR (95%CI)
**Overweight** **+** **obesity combined**				
English-speaking (ref)	1.0	1.0	1.0	1.0
European	**1.46 (1.18, 1.81)**	1.10 (0.82, 1.47)	1.31 (0.98, 1.75)	1.08 (0.80, 1.45)
Middle Eastern	**1.30 (1.03, 1.64)**	**1.42 (1.11, 1.81)**	**1.38 (1.10, 1.73)**	**1.29 (1.08, 1.52)**
Asian	0.89 (0.72, 1.10)	0.83 (0.69, 1.00)	1.03 (0.90, 1.18)	0.94 (0.78, 1.12)
**Obesity**				
English-speaking (ref)	1.0	1.0	1.0	1.0
European	1.05 (0.58, 1.89)	1.54 (0.83, 2.86)	**2.14 (1.42, 3.23)**	0.79 (0.41, 1.55)
Middle Eastern	**1.61 (1.16, 2.24)**	**1.49 (1.02, 2.16)**	**1.65 (1.03, 2.64)**	1.24 (0.74, 2.06)
Asian	0.78 (0.48, 1.27)	0.72 (0.44, 1.16)	1.00 (0.71, 1.39)	**0.55 (0.35, 0.86)**
**WHtR** **≥** **0.5**				
English-speaking (ref)	1.0	1.0	1.0	1.0
European	**1.71 (1.20, 2.42)**	1.13 (0.62, 2.06)	**1.59 (1.11, 2.29)**	0.62 (0.32, 1.19)
Middle Eastern	1.34 (0.95, 1.88)	**1.90 (1.29, 2.79)**	**1.68 (1.18, 2.40)**	**1.42 (1.08, 1.90)**
Asian	0.95 (0.66, 1.36)	0.68 (0.41, 1.12)	1.13 (0.87, 1.48)	0.76 (0.56, 1.03)

English-speaking reference category for each survey year; adjusted for sex, age, SES tertile (low, middle, high), residence (rural, urban); bold values are significant at *p* < 0.05

### Temporal trends in adiposity outcomes by language background

Table 4 presents adjusted PRs for each adiposity outcome by language background and survey year, adjusted for sex, residence, SES, and school level. The trend PR represents the magnitude of change over time and shows there were significant changes in the prevalence of overweight and obesity combined and WtHR ≥ 0.5, but not obesity rates. Between 1997 and 2015, the prevalence of overweight and obesity combined has increased among children from English-speaking (PR for trend: 1.06; 95%CI: 1.02, 1.09), Middle Eastern (PR for trend: 1.14; 95%CI: 1.05, 1.24), and Asian (PR for trend: 1.14; 95%CI: 1.05, 1.24) backgrounds. The PR trends indicate, between 1997 and 2015, the prevalence WtHR ≥ 0.5 increased by 21% (95%CI: 1.13, 1.31) and 35% (95%CI: 1.16, 1.56) in children from English-speaking and Middle Eastern language backgrounds, respectively.

**Table 4:** Temporal trends in adiposity outcomes by language background (PR, 95%CI), 1997 referent year, Australia; 1997–2015

Adiposity outcomes	1997 (ref)	2004 PR (95%CI)	2010 PR (95%CI)	2015 PR (95%CI)	Trend PR (95%CI)	PR for trend
**Overweight** **+** **obesity combined**						
English	1.0	**1.14 (1.03, 1.27)**	1.08 (0.98, 1.20)	**1.23 (1.11, 1.37)**	**1.06 (1.02, 1.09)**	**0.001**
European	1.0	0.86 (0.62, 1.21)	1.02 (0.70, 1.47)	0.96 (0.67, 1.36)	1.00 (0.89, 1.12)	0.984
Middle Eastern	1.0	1.26 (0.95, 1.69)	1.27 (0.97, 1.65)	**1.50 (1.17, 1.92)**	**1.14 (1.05, 1.24)**	**0.002**
Asian	1.0	1.11 (0.84, 1.47)	**1.34 (1.03, 1.74)**	**1.45 (1.09, 1.93)**	**1.14 (1.05, 1.24)**	**0.002**
**Obesity**						
English	1.0	1.13 (0.93, 1.37)	0.94 (0.77, 1.15)	1.22 (.099, 1.50)	1.05 (0.98, 1.12)	0.197
European	1.0	1.63 (0.71, 3.72)	**2.11 (1.03, 4.29)**	1.01 (0.42, 2.42)	1.11 (0.92, 1.42)	0.359
Middle Eastern	1.0	0.98 (0.65, 1.48)	1.00 (0.65, 1.53)	1.19 (0.77, 1.83)	1.06 (0.92, 1.23)	0.415
Asian	1.0	0.96 (0.47, 1.95)	1.19 (0.66, 2.16)	0.96 (0.50, 1.81)	1.02 (0.84, 1.23)	0.861
**WHtR** **≥** **0.5**						
English	1.0	**0.53 (0.44, 0.63)**	0.95 (0.79, 1.14)	**1.33 (1.12, 1.57)**	**1.21 (1.13, 1.31)**	**<0.001**
European	1.0	**0.37 (0.19, 0.71)**	0.96 (0.60, 1.54)	0.56 (0.29, 1.09)	0.89 (0.72, 1.10)	0.282
Middle Eastern	1.0	0.76 (0.46, 1.24)	1.40 (0.94, 2.09)	**1.86 (1.28, 2.71)**	**1.35 (1.16, 1.56)**	**<0.001**
Asian	1.0	**0.36 (0.21, 0.61)**	1.08 (0.70, 1.65)	1.04 (0.64, 1.70)	1.17 (0.99, 1.39)	0.062

Prevalence ratios adjusted for sex, age, SES tertile (low, middle, high), residence (rural, urban); bold values are significant at p < 0.05

## Discussion

This is the first study to examine the distribution of adiposity outcomes among Australian children from CALD backgrounds over an 18-year period. The most consistent finding across survey years was the high prevalence of adiposity outcomes among children from Middle Eastern language backgrounds, compared with children from English-speaking backgrounds. Children from European language backgrounds generally had higher prevalence and children from Asian language backgrounds had lower prevalence, compared with children from English-speaking backgrounds. Temporally, there have been significant increases in the prevalence of overweight and obesity combined and abdominal obesity in children from English-speaking, Middle Eastern, and Asian backgrounds with larger increases observed among the latter two groups.

Our findings are consistent with similar studies in Australia [3–5] and internationally that indicate obesity is disproportionally prevalent among children from CALD backgrounds, compared with children from host nations, including the UK [27], the US [28, 29], and European countries [30, 31]. In multi-cultural societies, understanding what factors contribute to differences in weight status among immigrant children is an important yet complex task. The life course approach attempts to integrate biological and social risk processes [7]. The processes of acculturation (adopting host culture) and enculturation (retaining traditional heritage) potentially play important roles in health-related outcomes in CALD communities [6]. Studies of immigrant population, including children, show that higher levels of acculturation are consistently associated with poorer health outcomes including adiposity outcomes [32].

Limited proficiency in a host nation language may disadvantage children’s health by limiting understanding, awareness, access, and participation in health promotion initiatives. A recent qualitative study of Australian migrant parents found a low level of obesity literacy, defined as individuals and communities’ knowledge, skill and ability to understand the importance of maintaining a healthy weight, recognised the impact of weight-related behaviours on health and being able to address weight-related health issues [33]. Other research among immigrant families indicates that obesity is perceived as a Western issue, and one that does not affect them or their children [34, 35]. Our findings showing the consistently high prevalence of obesity among children of Middle Eastern background and the increasing prevalence of obesity among children of Asian background highlights the importance of obesity prevention in these communities, possibly through culturally sensitive and tailored public health interventions to improve obesity-specific health literacy and health practices.

The noticeable increasing trend in adiposity outcomes among children from Asian backgrounds is consistent with previous studies, which showed a rapid upward assimilation of Asian immigrant’s BMI to the host countries over the generation [36–38]. Further, an Australian study showed that Asians who immigrated as a child/adolescent were more likely than adult immigrants to be overweight/obese [39]. This could be due to early exposure to Western culture associated with quicker adoption of Western lifestyles, which can affect body composition during childhood and adulthood, with long-lasting consequences on health [39].

Abdominal obesity is associated with an increased risk of cardiometabolic disease [28, 40], therefore the increasing prevalence among children from Asian language backgrounds in this study is particularly concerning. People from Asian backgrounds have a higher genetic predisposition to type-2 diabetes [41] because Asians tend to have a higher percentage of body fat and a worse profile of abdominal obesity, compared to Europeans with similar BMI, which predisposes Asians to insulin resistance at lower degrees of obesity [42, 43]. Asians are the largest migrant group in Australia, hence the importance to monitor their adiposity outcomes and consider culturally appropriate obesity prevention to prevent future cardiometabolic risks.

Similarly, the consistently high prevalence and increasing trend in adiposity outcomes among children from Middle Eastern language backgrounds is also concerning, and this has been shown in previous studies [44]. The recent Non-Communicable Disease Risk Factor Collaboration Study [1] has shown that obesity among children in Middle Eastern countries are amongst the highest in the world (around 20%), partially as a result of rapid economic transition in Middle Eastern countries. The aetiology of adiposity is complex, however diet and physical activity are recognised as key drivers behind the global increase in obesity [45]. In Australia, children from Middle Eastern language backgrounds report a higher prevalence of junk food [46] and soft drink consumption than their English-speaking counterparts [47]. Additionally, children from Middle Eastern language backgrounds, compared with English-speaking peers, have lower cardiorespiratory fitness, lower physical activity participation, and higher screen time [3, 6, 10, 14]. A recent Australian study showed that parents from Middle Eastern backgrounds generally encourage healthy behaviours for their children but report frequent exemptions [48], which may represent an indulgent or permissive style of parenting that has been consistently associated with a higher BMI [49].

Although there has been a substantial multi-sectorial investment to reduce child obesity in NSW through a succession of state plans, policies, and programmes [50–53], the consistently higher prevalence of adiposity outcomes among children with Middle Eastern language backgrounds in this study suggests these investments have not reached these children. A contributing factor may be because interventions that specifically target Australian children from CALD backgrounds appear limited [54, 55].

There are several strengths of this study. Firstly, the representativeness of our sample allows the generalisability of our findings to NSW children from Asian, European, and Middle Eastern language backgrounds. Second, the methodology was consistent across the four survey years, and adiposity outcomes based on anthropometry (rather than self-report). The use of a single item question to determine children’s CALD background may have limitations, however this question comes from the national census [23] and is used to determine proficiency in spoken English, which may be an indicator of a person’s ability to participate effectively in Australian society, including accessing government and other services.

Although our sample size prohibited classifying children into specific CALD groups, the three discreet language categories we used to represent the most prevalent immigrant groups in NSW [12]. A limitation of the broader language categories is the attenuation of cultural and ethnic diversity. For example, Asians included children from Indian, Chinese, and Mongolian backgrounds, whose genetic predisposition, cultural practices, and parenting styles relevant to childhood obesity may differ noticeably. Further, relying on language spoken at home for classifying cultural background may lead to misclassification; for example, English-speaking Indians and Singaporeans would not be classified as ‘Asian’ by the current definition. Finally, the question was designed to measure a person’s first language, however children of immigrants who have been in Australia for many years may report English as the language spoken most at home.

Australia is one of the most multicultural countries in the world and information on the distribution of adiposity outcomes among children from CALD communities living in Australia is important to the design and delivery of obesity prevention initiatives within these communities. There has been considerable research into understanding cultural norms associated with food, diet, and physical activity in CALD communities in Australia [56–58], however in NSW, the prevalence of overweight and obesity and abdominal obesity is high among CALD children, and has increased over time. This suggests that there is a greater scope in understanding, developing, and implementing interventions across the early life-course of CALD children. The WHO life course recommendations [59] provide the scaffolding for governments to address child obesity within CALD communities, including interventions for women’s health preconception to address environmental factors within communities with high CALD population that support or undermine the availability and access to healthy food choices and safe spaces to be physically active.
